# Machine learning models reveal distinct disease subgroups and improve diagnostic and prognostic accuracy for individuals with pathogenic *SCN8A* gain-of-function variants

**DOI:** 10.1242/bio.060286

**Published:** 2024-04-24

**Authors:** Joshua B. Hack, Joseph C. Watkins, Michael F. Hammer

**Affiliations:** ^1^ BIO5 Institute is Keating Research Building, 1657 E Helen Street, University of Arizona, Tucson, AZ 85721, USA; ^2^ Department of Mathematics, University of Arizona, Tucson, AZ 85721, USA; ^3^ BIO5 Institute, Neurology Department, University of Arizona, Tucson, AZ 85721, USA

**Keywords:** Pediatric epilepsy, Rare disease, Genetic epilepsy, Clinical phenotype, Unsupervised learning, Patient registry

## Abstract

Distinguishing clinical subgroups for patients suffering with diseases characterized by a wide phenotypic spectrum is essential for developing precision therapies. Patients with gain-of-function (GOF) variants in the *SCN8A* gene exhibit substantial clinical heterogeneity, viewed historically as a linear spectrum ranging from mild to severe. To test for hidden clinical subgroups, we applied two machine-learning algorithms to analyze a dataset of patient features collected by the International SCN8A Patient Registry. We used two research methodologies: a supervised approach that incorporated feature severity cutoffs based on clinical conventions, and an unsupervised approach employing an entirely data-driven strategy. Both approaches found statistical support for three distinct subgroups and were validated by correlation analyses using external variables. However, distinguishing features of the three subgroups within each approach were not concordant, suggesting a more complex phenotypic landscape. The unsupervised approach yielded strong support for a model involving three partially ordered subgroups rather than a linear spectrum. Application of these machine-learning approaches may lead to improved prognosis and clinical management of individuals with *SCN8A* GOF variants and provide insights into the underlying mechanisms of the disease.

## Teaser

**Summary:** Application of machine learning methods to data collected by the International SCN8A Registry identifies clinically meaningful subgroups within the broad spectrum of disorders suffered by patients possessing gain-of-function variants.

## INTRODUCTION

Rare diseases are estimated to affect approximately 1 of every 10 individuals in the human population. The low incidence coupled with the relatively recent introduction of genetic testing often means there are too few known cases upon which to establish effective protocols for diagnosis, prognosis, and treatments. In addition, the wide phenotypic spectrum often associated with many rare diseases makes it challenging to identify targeted treatment strategies (Imbrici et al., 2016). Patient registries play an indispensable role in filling gaps in the literature for rare and newly discovered diseases that have few published case reports or cohort studies. The addition of patient information coupled with advancements in computational sciences and machine learning (ML) to streamline the process of organizing, transforming, and analyzing large amounts of data hold promise for more efficient pipelines moving from gene discovery to precision medicine (Knowles et al., 2022). By using more traditional statistical methods alongside ML models, researchers can compare treatment efficacy (Cutter et al., 2019; Lee et al., 2017), predict survival probability (Mahadevan et al., 2018) and health risk factors (Taushanov et al., 2021), and improve diagnosis (Bica et al., 2021; Hack et al., 2023; Zou and Ma, 2020). These methods facilitate construction of clinical tools to describe disease and predict a patient's optimal treatment based on both genotypic and phenotypic characteristics, providing the possibility for personalized medicine.

An example of the success of the application of these methods to registry data is the case of SCN8A-epilepsy and related disorders (Knowles et al., 2022). Patients with pathogenic variants in the *SCN8A* gene, encoding the voltage-gated sodium channel Na_V_1.6, exhibit substantial clinical heterogeneity, with phenotypes ranging from neurodevelopmental delays with or without seizures, to benign familial epilepsy, to a continuum of mild to severe development and epileptic encephalopathy (DEE) (Cutts et al., 2022; Hammer et al., 2023; Johannesen et al., 2022; Talwar and Hammer, 2021). The *SCN8A* gene was discovered to cause pediatric epilepsy in 2012 (Veeramah et al., 2012), and an online registry was established in 2014 that now contains an extensive dataset encompassing medical, genetic, developmental, and comorbidity information for over 400 international cases (Andrews et al., 2023; Chung et al., 2023; Encinas et al., 2019). Given the wide phenotypic spectrum and numerous pathogenic variants in the gene, both with loss-of-function (LOF) and gain-of-function (GOF) effects, clinicians have been challenged to provide accurate diagnosis, prognosis, and a course of effective treatment. Recent advances have made it clear that the LOF and GOF subtypes differ in biological terms given the different effects LOF and GOF variants have on Na_V_1.6 function and differential response to antiseizure medications (ASMs) (Hack et al., 2023; Johannesen et al., 2022; Liu et al., 2019; Wagnon et al., 2016). Hack et al. (2023) developed a predictive modeling approach to classify GOF and LOF variants based on clinical features present at initial diagnosis. This was an important step given that *in vitro* studies to infer channel function are not feasible in the clinic. Despite these gains, currently there are no guidelines for best practices in treating SCN8A-related epilepsy.

An important unanswered question is whether individuals across the phenotypic spectrum associated with variants with GOF effects can be subdivided into subgroups that differ in response to ASMs. Indeed, individuals carrying different recurrent GOF variants are known to vary in disease course, and possibly in response to ASMs (Chung et al., 2023; Hammer et al., 2023). In this study, we analyze the International SCN8A Registry data representing 253 cases to identify key features present early in disease progression and construct a series of predictive models to classify individuals possessing GOF variants as mild, moderate, or severe cases. Each model is assessed for accuracy via confusion matrices or mean error to test the hypothesis that the population of patients possessing GOF variants is composed of three distinct groups.

## RESULTS

### Loss-of-function exclusion

Application of the LOF Classifier (Hack et al., 2023) identified 180 of 253 individuals in the International SCN8A Patient Registry (Andrews et al., 2023; Encinas et al., 2019) as having a high probability of possessing a pathogenic GOF *SCN8A* variant. These 180 individuals constituted the dataset used in this study, and justification for inclusion of the variant is provided in Table S1. The average and standard deviation of age at onset in this subpopulation was 3.6±2.86 months and all individuals reported experiencing seizures. The average developmental quotient was 36.8±39.87 with 81% reporting developmental delay. The frequencies of seizure types are described in Table 1. Of 115 variants, 87 were singletons, 18 were doubletons, and 10 were reported in at least three individuals. 100 variants (119 individuals) were inferred to be GOF (Hack et al., 2023) and 15 of these variants (61 individuals) were known to be GOF based on electrophysiological data.

**Table 1.**
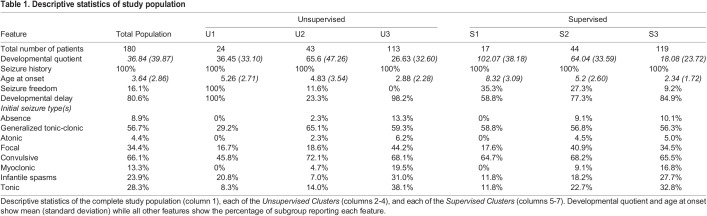
Descriptive statistics of study population

### Cluster assignment

**Fig. 1.**
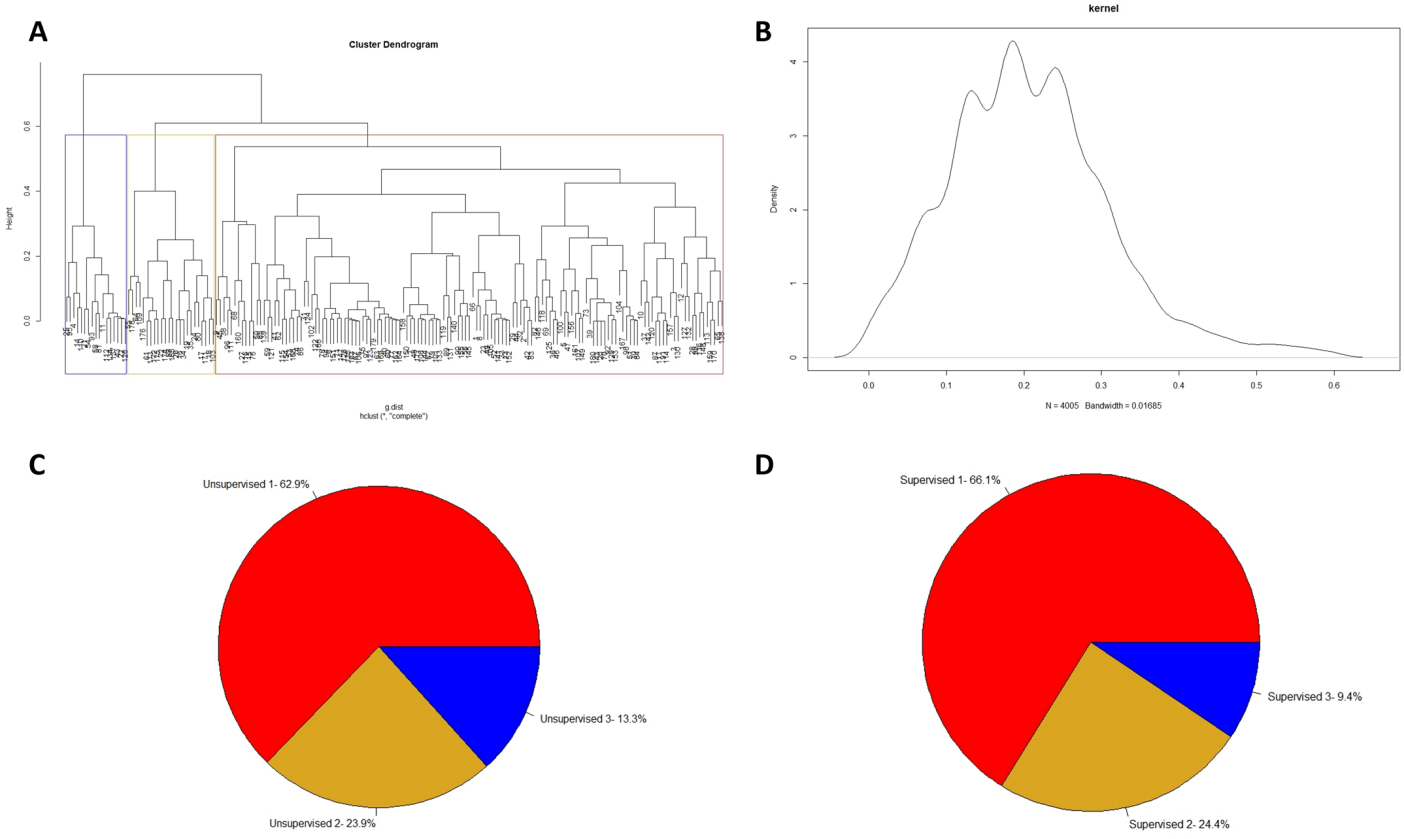
**The *SCN8A* GOF population can be split into three groups, with strong imbalance.** (A) Hierarchical cluster analysis showing the cutoff for optimal number of groups. Hierarchical clustering used the complete agglomeration method for the optimal dendrogram. Cluster U1 (blue, *n*=24), cluster U2 (yellow, *n*=43), and cluster U3 (red, *n*=113) are marked in their respective colored boxes. (B) Kernel density plot. Peaks around 0.2 show 3 optimal clusters. (C) Distribution of patients in clusters assigned by hierarchical clustering in the *Unsupervised Approach*. (D) Distribution of patients in clusters assigned by clinical and researcher guidelines in the *Supervised Approach.*

Unsupervised clustering analysis using partitioned around medoid (PAM) clustering showed the optimal number of clusters in this dataset was three. We denote these clusters U3, U2, and U1. This finding is consistent with the common understanding that mild, moderate, and severe groups exist in the *SCN8A* GOF population, as shown in previous work (Hack et al., 2023) (Fig. 1A). Testing of the optimal number of clusters using kernel density estimation further supported this finding (Fig. 1B). The average age at onset and developmental quotient (DQ; see Materials and Methods for definition) were used to determine an order to the assigned clusters, and it was found that there was an increase in age at onset from cluster U3 to cluster U1 (Table 1). This suggested cluster U3 correlated to a severe phenotype, cluster U2 to a moderate phenotype, and cluster U1 to a mild phenotype. Notably, developmental quotient did not follow this trend and was 26.6±32.6 and 36.5±33.1 in clusters U3 and U1, respectively, while being higher in cluster U2 at 65.6±47.3; (a quotient of 100 is considered neurotypical). The distribution of individuals in each category showed a strong imbalance favoring cluster U3 (Fig. 1C). Three clusters were also assigned as part of a *Supervised Approach* by using clinical severity cutoffs. These clusters were considered severe, moderate, and mild DEE and we denote these clusters S3, S2, and S1, respectively. Similar to the *Unsupervised Approach*, there was a strong imbalance in cluster size favoring S3 (Fig. 1D).

### Dimension reduction

**Fig. 2.**
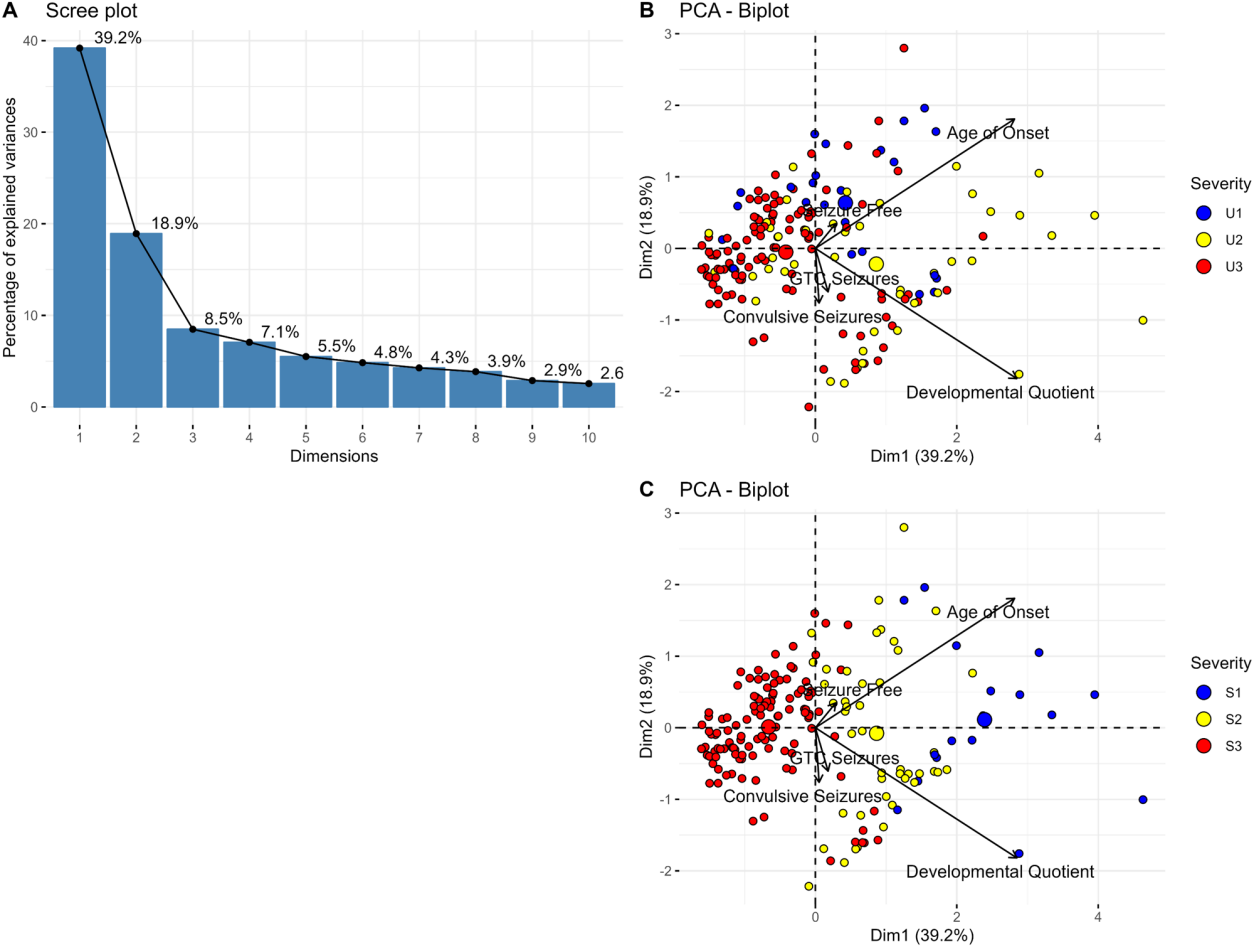
**Dimension reduction of *SCN8A* GOF population shows limited separation between groups.** (A) Scree plot showing percentage of variance explained in each dimension using *Unsupervised Clusters*. (B) Two-dimensional PC plot showing distribution of cluster U3 (red, *n*=113), cluster U2 (yellow, *n*=43), and cluster U1 (*blue, n*=24). The five features with highest contribution to variance are shown as vectors in *black*. (C) Two-dimensional PC plot showing distribution of cluster S3 (red, *n*=119), cluster S2 (yellow, *n*=44), and cluster S1 (blue, *n*=17). The five features with highest contribution to variance are shown as vectors in black.

Principal component (PC) analysis on the data shows that 66.6% of the variance in the dataset was explained using the first three dimensions (Fig. 2A). A two-dimensional PC plot showed developmental quotient and age at onset having the greatest contribution to the variance in the dataset (Fig. 2B). It was apparent that there was a significant overlap in the groups assigned by the *Unsupervised Approach*. However, using a three-dimensional PC plot showed increased differentiation between groups (Fig. S1). While there were distinct centroids for each group, there was high variability within cluster U2. The cluster U3 group appeared to be the most uniform, with the majority being located to the left of the y-axis with its centroid close to the x-axis. Across the three groups, there was a general trend of increasing age at onset from cluster U3 to U1.

The *Supervised Approach* showed distinction between the three groups in two dimensions (Fig. 2C). Individuals in cluster S3 were centered to the left of the y-axis and above the x-axis. Individuals in the cluster S2 or cluster S1 group were centered progressively more positively along the x-axis with a clearer division between the two phenotypic groups than seen in the *Unsupervised Approach*. Most individuals in cluster S1 were also above the x-axis, suggesting better development and lower rates of bilateral tonic clonic (BTC) and convulsive seizures at initial presentation. The distinction between each group was more visible in a three-dimensional PC plot (Fig. S2).

### Penalized ordinal logistic regression model

A penalized ordinal logistic regression model (p-ORM) was constructed for both the *Unsupervised* and *Supervised Approaches*. Both approaches used *k*-fold cross-validation where *k*=5. In the *Unsupervised p-ORM,* the misclassification error across five iterations was 0.105±0.04. Tuning of the model resulted in seizure freedom, DQ and developmental delay, age at onset, infantile spasms, convulsive, myoclonic, absence, focal, and tonic seizures being selected (Table 2). Following penalization, the optimal tuning parameter (best lambda index) was λ=18 and a confusion matrix was constructed using this parameter, resulting in an error of 0.089 (Table 3). The *Supervised p-ORM* resulted in a misclassification error of 0.144±0.04. Features that were selected by tuning the model included age at onset, seizure freedom, DQ, tonic, convulsive, and BTC seizures (Table 2). Penalization resulted in λ=14 and a confusion matrix with an error of 0.139 (Table 3).

**Table 2.**
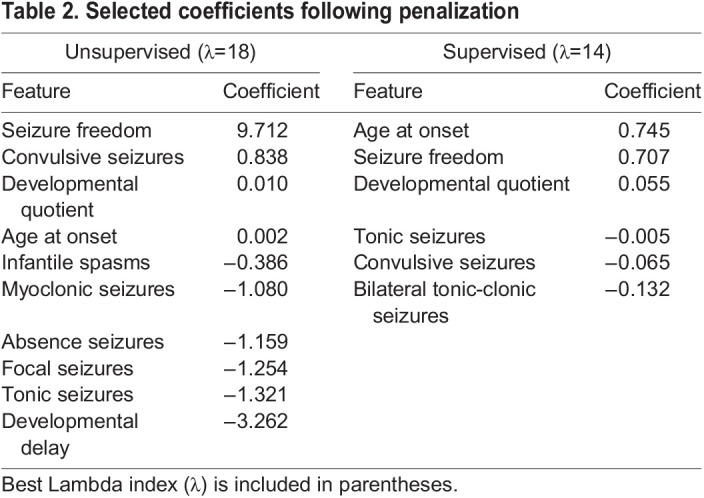
Selected coefficients following penalization

**Table 3.**
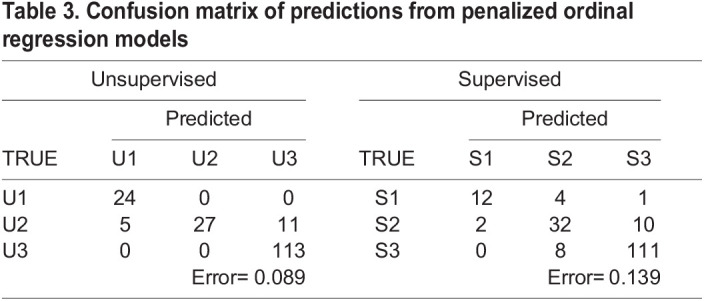
Confusion matrix of predictions from penalized ordinal regression models

### Stacked meta-learner

Both datasets were analyzed using a stacked meta-learner (Stacked) three times. The dataset was first sampled in five separate ways before a random forest model was trained and tested using *k*-fold cross validation where *k*=4 using the methods described in (Desprez et al., 2022). The predicted outcome classifications from each of the sampling methods were used as training features in an ordinal logistic regression model, while the probabilities were used in a linear regression model as a meta-learner. In the *Unsupervised Stacked Model*, the mean absolute error (MAE) was 0.06±0.01 and 0.04±0.03 for, respectively, the outcome classification and probability from the random forest model (Table 4). The root-mean-square error (RMSE) was 0.16±0.01 and 0.15±0.07 and the percentage of correct classification (PCC) 94.8±0.8% and 95.2±2.70% for classification and probability, respectively. In the *Supervised Stacked Model*, performance resulted in MAEs of 0.37±0.09 and 0.35±0.07, RMSEs of 0.54±0.07 and 0.52±0.08, and PCCs of 81.0±4.58% and 81.0±1.00% for classification and probability, respectively (Table 4).

**Table 4.**
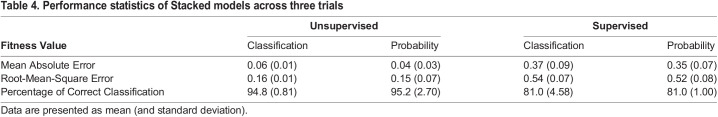
Performance statistics of Stacked models across three trials

### External validation

**Fig. 3.**
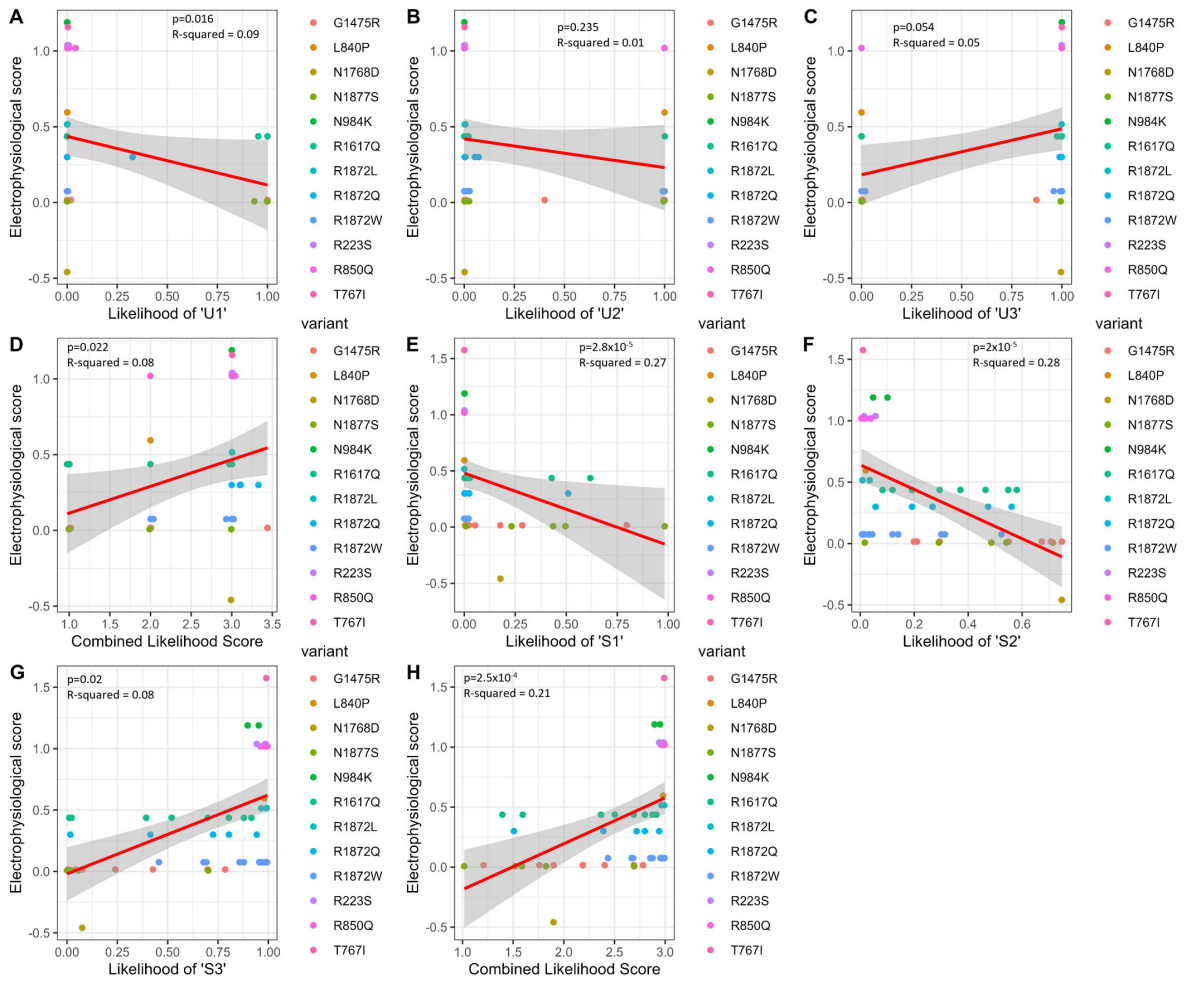
**Linear regression between group probability and variant electrophysiological score suggests biological relevance.** Linear regression between group probability and electrophysiological score for (A-D) *Unsupervised Stacked* and (E-H) *Supervised p-ORM*. Electrophysiological score as a function of probability of classification as (A) U1, (B) U2, (C) U3, (D) combined probability score from *Unsupervised Stacked* model, (E) S1, (F) S2, (G) S3, and (H) combined probability score from the *Supervised p-ORM* model. Variants included for *Unsupervised Stacked*: p.R223S (*n*=2), p.T767I (*n*=1), p.L840P (*n*=1), p.R850Q (*n*=7), p.N984K (*n*=2), p.G1475R (*n*=7), p.R1617Q (*n*=8), p.N1768D (*n*=1), p.R1872L (*n*=2), p.R1872Q (*n*=4), p.R1872W (*n*=11), p.N1877S (*n*=5). Variants included for *Supervised p-ORM:* p.R223S (*n*=3), p.T767I (*n*=1), p.L840P (*n*=1), p.R850Q (*n*=9), p.N984K (*n*=2), p.G1475R (*n*=7), p.R1617Q (*n*=8), p.N1768D (*n*=1), p.R1872L (*n*=2), p.R1872Q (*n*=5), p.R1872W (*n*=12), p.N1877S (*n*=5).

To determine whether the *Unsupervised Stacked* and *Supervised p-ORM* have biological and clinical relevance, each model was validated using external data (Johannesen et al., 2022). Biological relevance was tested using variants that have undergone electrophysiological studies and have an associated score based on six distinctive features. Linear regressions using values from the *Unsupervised Stacked* model showed a significant correlation against a test of no correlation between cluster U3 and electrophysiological score and the combined score and electrophysiological score (*P*-value=0.016 and 0.022, respectively, and adjusted R-squared=0.094 and 0.084, respectively) (Fig. 3A-D). Additionally, the probability of being cluster U1 showed a nearly significant relationship with electrophysiological score (*P*-value=0.054; adjusted R-squared= 0.055). R1872W has been suggested to be an outlier regarding its electrophysiological score and phenotypic severity, potentially due to its difference in peak current from R1872Q, which was not included in the calculation of electrophysiological score (Chung et al., 2023; Johannesen et al., 2022). To account for this, regression was again performed excluding R1872W. All *P*-values were found to decrease while R-squared values increased, with cluster U3, cluster U1, and Combined showing significant correlation against a test of no correlation (*P*-value=0.002, 0.010, and 0.004, respectively, and adjusted R-squared=0.207, 0.141, and 0.181, respectively).

In the *Supervised p-ORM*, linear regressions of all four severity scores show significant relationships with electrophysiological score with *P*-values of 2.84×10^−5^, 1.95×10^−5^, 0.022, and 2.48×10^−4^ for cluster S3, S2, S1, and Combined, respectively, and adjusted R-squared of 0.266, 0.276, 0.077, and 0.208, respectively (Fig. 3E-H). Further validation of the *Supervised p-ORM* was conducted using a dataset of individuals with clinical decisions made regarding their severity (Johannesen et al., 2022). The *Supervised p-ORM* was used to predict on this dataset, using the same features. Prediction resulted in a mean error of 0.209.

## DISCUSSION

The application of machine-learning techniques resulted in statistical support for three distinct patient subgroups within the wide phenotypic spectrum associated with *SCN8A* GOF variants. Using features with severity cutoffs that were determined by clinical conventions, the *Supervised Approach* categorized individuals into three groups that supported the historical view of Mild, Moderate, and Severe DEE subgroups. The *Unsupervised Approach* confirmed the presence of three disease GOF subgroups using a strictly data-driven strategy (i.e. without the bias of prior clinical interpretation). However, the distinguishing features of the three subgroups were not concordant between the two approaches. A key finding of the Unsupervised Approach is the clinical separation of epileptic and developmental encephalopathies. In the following sections we discuss the advantages and disadvantages of each approach and the significance of the finding of distinct disease subgroups.

Both the *Unsupervised* and *Supervised Approaches* were modeled using penalized ordinal logistic regression to identify features that were critical to distinguishing the three clusters and again using a stacked meta-learner to account for sample size imbalance within groups. In the *Unsupervised Approach* it was found that both modeling methods performed well in classifying individuals, with the average error being 0.11 and 0.04 for the p-ORM and stacked models, respectively (Tables 3-4). The primary concern for both the *Unsupervised Stacked* and *Unsupervised p-ORM* is the potential for over-reliance on seizure freedom as a predictive feature. While the p-ORM shows a strong impact from seizure freedom (Table 2), the effect is limited in the *Unsupervised Stacked*. This, taken together with the improvement in performance metrics, makes the stacked meta-learner the preferred choice.

In the *Supervised Approach*, only the p-ORM performed well enough to be considered viable in further analysis. This model recorded an average error of 0.144 across the five folds in cross validation and a final error of 0.139 after penalization (Table 3) compared to the average MAE of 0.35 observed in the Stacked model (Table 4). In this approach, the complexity of the Stacked meta-learner may lead to a decrease in overall performance. As such, the *Supervised Approach* is best modeled using the penalized ORM. This model selected age at seizure onset as its primary feature contributing to predicted outcome, as expected from the original classification guidelines (Table 2).

Both the *Unsupervised* and *Supervised Approaches* were tested for biological relevance following the success of modeling. In the case of the *Unsupervised Stacked* model, the predicted probabilities of an individual being in cluster U3 and U1, as well as a combined probability score across all three clusters, were shown to have a significant linear relationship with electrophysiological score calculated for highly recurrent *SCN8A* variants (Fig. 3). Similarly, the *Supervised p-ORM* showed significant relationships between all categories and variant-specific electrophysiological score (Fig. 3). The variant electrophysiological score has previously been shown to correlate with clinical severity (Chung et al., 2023; Johannesen et al., 2022). While this is a simplified surrogate parameter, the congruence between these electrophysiological scores and predicted outcome lends additional support to the validity of these classifications.

Additional relevance testing was conducted on the *Supervised p-ORM* using an externally curated dataset (Johannesen et al., 2022) to test the performance of the model. In this case, the error of the predictive model increased from 0.139 to 0.209. This decrease in performance is expected, as the dataset used for external validation collected data in a different format than the data used in this study. For example, developmental quotient was not available for individuals in the external dataset and was collected as categorical values of intellectual and developmental disorder, rather than a continuous variable. Individuals were given generalized values in instances of data collection discrepancies, which can lead to underfitting in a ML model.

### Discordance in clusters

The results of the *Unsupervised Approach* to identifying and assigning clusters provide a novel method for classifying individuals. In the case of the *Supervised Approach*, clusters correspond to Mild DEE, Moderate DEE, and Severe DEE as described (Hammer et al., 2023). As expected, DQ follows a trend of decreasing from cluster S1 to S3. Seizure-free individuals are present in all three clusters. Strikingly, the *Unsupervised Approach* is not concordant with these results. In cluster U1, DQ is lower in both cluster U2 and cluster U3. This contrasts with age at onset is higher than cluster U2 but lower in cluster U3. Moreover, *all* individuals experience seizure freedom in cluster U1. cluster U2 is characterized by no to moderate developmental delay with an earlier average age at onset than cluster U1. cluster U3 shows severe developmental delay and exceedingly early age at onset with no individuals experiencing seizure freedom. Those who attained prolonged seizure freedom likely benefitted from effective treatment with ASMs. It should be noted that SeLFIE [self-limited(familial) infantile epilepsy] is an additional phenotype that was not observed in the Registry.

The marked non-concordance between the two approaches challenges the conventional understanding of the *SCN8A* GOF population as an ordered spectrum from mild to severe DEE. Taken as a whole, cluster U1 experiences the latest average age at seizure onset, while also having few relative instances of more aggressive seizure etiologies such as myoclonic seizures. Also, this group consistently achieves seizure freedom. These characteristics tend to align with the expectation for a mild DEE, but the severe developmental delay indicated by a low average DQ conflicts with this understanding under the presently used classification of SCN8A epilepsy. However, cluster U2 is characterized by a moderate average age at onset with a low seizure-freedom rate. These seizure characteristics are consistent with a moderate DEE, but fewer than a quarter (10/43 individuals) report developmental delay within this group, once again being at odds with the current moderate DEE classification. cluster U3 is the only group largely concordant with its expected counterpart S3 in the *Supervised Approach* in both DQ and age at onset. Notably, this group reports zero incidents of seizure freedom compared to the 11 incidents of seizure freedom reported in cluster S3 (Table 1).

The notion that individuals with *SCN8A* GOF do not neatly fit a linear spectrum of increasingly severe DEE is not entirely unexpected. The distinction between mild and moderate DEE has been a consistent challenge for clinicians, leading to these two categories often being collapsed (Hammer et al., 2023). As such, we hypothesize that the three clusters identified using the *Unsupervised Approach* are a partially ordered subgroup set where cluster U1 is primarily a developmental encephalopathy (DE), cluster U2 is primarily an epileptic encephalopathy (EE), and cluster U3 is a DEE. Given this hypothesis, it is predicted that individuals in the DE population typically would experience developmental delay prior to seizure onset, while those in EE would experience seizure onset prior to developmental delay. Those in DEE category would be expected to have clinical diagnoses of both seizure onset and developmental delay nearly simultaneously within the first few months of life.

Our results are in line with previous studies demonstrating correlations between specific pathogenic variants and severity outcome (Chung et al., 2023). For example, the most debilitating variants – p.R850Q and p.R1872W – were classified as DEE 90.5% of the time (19/21), while variants that are associated with milder clinical outcomes such as p.G1475R and p.N1877S were classified as either DE or EE 83.3% of the time (10/12). This provides a second line of support for a partially-ordered model in which DEE is the most severe subgroup, with similar degrees of severity for DE and EE. Thus, a strictly ordinal model is unlikely to be the most appropriate for classifying individuals with GOF variants. As more phenotypic data become available from individuals with highly recurrent variants, our model of subgroup classification may reveal a deeper understanding of the underlying mechanisms of the disease. In addition, the mechanistic properties of different GOF variants as inferred from *in vitro* electrophysiological studies may correlate with particular disease subgroups (Chung et al., 2023).

### Implications for treatment

This study provides a critical insight for clinicians to use in assessing a patient's prognosis. The two approaches used here result in models that perform with high accuracy and identify features that are important to understanding possible patient outcomes. These features are present early in the disease progression including age at seizure onset, developmental quotient, developmental delay, initial seizure type, and whether seizures have been controlled for at least 6 months. A strength of this model is that all features except prolonged seizure freedom can be assessed accurately in the first clinical visit following genetic testing. As such, it is believed that this model is particularly useful for improving prognosis. Not only do these models perform well based on features revealed early in life, but they have also been shown to have biological relevance as determined by regression on outcome probability versus electrophysiological score.

Given the two different approaches to classifying individuals, one model from each approach is proposed in aiding clinical decisions. In the case of the *Supervised Approach*, the penalized ordinal regression model performs with high accuracy and considers age at onset, DQ, and three seizure types as the most highly predictive features (Table 2). The low error and data requirement for this model makes it particularly well suited to clinical settings, especially compared to the *Supervised Stacked* model, which has a high error (Table 4). One important consideration for this model is the unbalanced cluster assignments that may lack power to detect some phenotype–phenotype associations. This is overcome by using the data-driven *Unsupervised Approach*. Not only is the bias limited in the *Unsupervised Approach*, both models using these clusters perform with lower error than the *Supervised p-ORM* (Tables 3 and 4). A shortfall of the *Unsupervised p-ORM* is its heavy reliance on seizure freedom (Table 2). Because of this, the *Unsupervised Stacked* model should be used if relying on this approach.

Further support of the hypothesis that the *SCN8A* GOF population can be categorized as DE, EE, or DEE will favor the use of the *Unsupervised Stacked* model over the *Supervised p-ORM*. This would have implications for effective treatment and would help to inform clinicians and caretakers of realistic treatment goals. In the case of DE, effective treatment may result in attainment of prolonged seizure freedom without notable improvements in developmental progression. On the other hand, despite seizures being more difficult to control for individuals with EE, some improvements in acquisition of developmental milestones may come with reduction in seizure burden. If an affected individual is classified as DEE, effective treatment may reduce seizure burden without achievement of seizure freedom or improvements in acquisition of developmental skills. As we acquire additional patient reports in support of this classification system, then the expectation is that this classification will become more standard among clinicians and thus become the supervised model for the next refinement in SCN8A classification.

### Caveats and significance

While both models are effective in accurately classifying individuals into their respective groups, these models are limited to the datasets available to them at training. As these models are further developed as more longitudinal data become available, it is expected that the models will continue to be refined and improve in accuracy. Currently, their utility is mainly as a tool to aid in clinical decisions. Despite these limitations, we believe that these models can be used in combination with the LOF Classifier (Hack et al., 2023) to provide rapid predictions of variant effect and expected patient subgroup. The results from the *Unsupervised Approach* also lay the foundation for a potential shift in the understanding of the phenotypic landscape of SCN8A Syndrome, with possible implications for disease mechanisms. The results from this work may help with developing more attuned machine learning models to better characterize the entire phenotypic spectrum, including both LOF and GOF variants with a variety of biophysical effects on the Na_V_1.6 channel. This will aid clinicians in making treatment decisions and provide more realistic expectations for caregivers of individuals with SCN8A-related disorders.

The construction of this predictive model provides the opportunity for further development of machine learning tools to assist in clinical settings. However, the field of functional prediction is still emerging and while these models show promise in a research capacity, they should not be used to make decisions on clinical management. Before widespread use of these models becomes a viable option in clinical settings, trials must be conducted to validate the results and test the robustness of these models. To emphasize, these models are not suitable replacements for clinical guidelines on managing SCN8A-related disorders, but rather tools to aid in research and may eventually be validated for clinical use. Clinicians who are interested in using these models should be aware of their limitations and use the results as only a part of their therapeutic strategies. Ultimately, these models are tools that may develop to higher magnitudes of utility but presently should be used with caution and awareness of the limitations and risks outside of a research setting.

## MATERIALS AND METHODS

### Data collection

Data for this study were collected by the International SCN8A Patient Registry (Andrews et al., 2023; Encinas et al., 2019). 253 responses during the period from January 2017 through December 2021 from consenting participants were considered for this study. Using this dataset, features were selected that are present and easily assessed in clinical settings at early stages of the disease: genetic variant, age at onset, seizure history, initial type(s) of seizure(s), current type(s) of seizure(s), developmental quotient (DQ), presence of developmental delay, and whether the patient had experienced a period of at least 6 months of seizure freedom. DQ was calculated by dividing developmental age by patient's age at the time of their registry submission multiplied by 100. Developmental age was calculated using 25 skills from the Denver II Developmental Test that are queried in the Registry. This quotient uses the 90th percentile for the age that a given skill is acquired by a neurotypical child. Initial and current seizure types were expanded from a list variable to a single binary variable for each seizure type.

### Inclusion criteria

Individuals were included in this study only if they possessed *SCN8A* variants with known or inferred gain-of-function (GOF) properties and were classified as pathogenic or likely pathogenic according to genetic reports, the ClinVar database, or according to American College of Medical Genetics (ACMG) guidelines (Richards et al., 2015). Variants known to have GOF properties from previous electrophysiological studies performed in heterologous expression systems were also included. Additional variants were included by utilizing the LOF Classifier (Hack et al., 2023) to categorize variants: those with a probability of loss of function (LOF) [prob(LOF)] <0.3 were considered GOF, while those with a prob(LOF) >0.3 were considered intermediate or true LOF variants and excluded from the study. Alternative methods exist for classifying variants as GOF or LOF (Bosselmann et al., 2023; Brunklaus et al., 2022; Heyne et al., 2020). After reviewing these alternatives, it was determined that the LOF classifier from Hack et al., 2023 was sufficient as justification for inclusion of variants in a clinically focused study. The alternative methods were determined to be better suited for evolutionary and comparative studies of sodium channels or in cases which a disease has limited available clinical data. Additionally, individuals with a pathogenic or likely pathogenic (but not benign or likely benign) variant at other loci associated with epilepsy were excluded from the study. The resulting dataset consisted of 180 individuals.

### Cluster assignment and data visualization

#### Unsupervised approach

Toward the goal of limiting clinician bias and discovering new phenotype–phenotype links, unsupervised cluster analysis and assignment was completed. Individuals were assigned clusters by using a dissimilarity matrix calculation with a generalized Gower formula resulting in an optimal number of three clusters, named U1, U2, and U3. This was determined using partitioned around medoid (PAM) clustering and further testing of these clusters using hierarchical clustering to validate these assignments. A range of 1–5 was tested for the optimal number of clusters and further validation of cluster assignments was conducted using density-based spatial clustering of applications with noise (DBSCAN). Analysis of each cluster's features suggested that cluster U1 associated with milder phenotypic outcome while cluster U3 associated with more severe outcomes, with cluster U2 being intermediate. These clusters were used as the response feature in each predictive model as an *Unsupervised Approach.*

#### Supervised approach

To ensure clinical relevance, assessment of patient health severity was determined by a clinical specialist. These group assignments were determined by age at onset, DQ, and a combination of initial seizure type and number of seizure types at initial presentation. The severity groups of mild (cluster S1), moderate (cluster S2), and severe (cluster S3) corresponded generally to the mild, moderate, and severe DEE categories described in (Hammer et al., 2023), respectively. There were no instances of the classification described as self-limited familial infantile epilepsy (SeLFIE) (Hammer et al., 2023). These severity groups were used as the response feature in each predictive model as a *Supervised Approach*.

### Predictive modeling

A series of predictive models were constructed using clusters assigned by both the *Unsupervised* and *Supervised Approaches.* In both approaches, penalized ordinal logistic regression (p-ORM) and a stacked meta-learner using a random forest classifier, an ordinal logistic regression classifier, and a linear regression model as described in (Desprez et al., 2022) (Stacked) were used to model the dataset. Each model used clusters as the response feature. In the case of the p-ORM and Stacked models, predictor features were age at onset, DQ, seizure freedom, initial seizure type(s), and developmental delay. The Stacked model accounts for imbalance between groups by constructing five random forest models using conventional sampling methods for contribution to predictive performance. These methods include no sampling, oversampling, undersampling, over/undersampling, and synthetic minority oversampling technique (SMOTE) sampling. The random forest probability and classification outputs of the selected sampling methods are then used as inputs in a linear and ordinal regression model, respectively, for a final classification.

For the p-ORM, *k*-fold cross validation was used with five folds to estimate an average error across the entire model. The optimal penalization parameter, λ, was calculated for both approaches. Similarly, the Stacked model utilized *k*-fold cross validation by splitting the training set into three folds. Each model was trained and tested on the holdout data and mean absolute error (MAE), root-mean-square deviation (RMSE), and percentages of correct classification (PCC) were used to evaluate the performance of each model. To ensure that the Stacked model's performance was not a result of favorable training holdouts, each Stacked model was run with three different seeds and the average MAE and RMSE from each run was calculated for a final performance value.

### External validation

To determine biological and clinical relevance, the Stacked model using the *Unsupervised Approach* and the p-ORM using the *Supervised Approach* were validated. Both models were validated for biological relevance by taking a subset (*n*=51) of the population possessing variants that have associated electrophysiological scores based on six distinctive features: half activation voltages of activation and inactivation curves, slopes of the activation and inactivation curves, maximum conductance, and persistent current reported in Johannesen et al., 2022. Recent work has shown these electrophysiological scores to be correlated with clinical severity (Chung et al., 2023). A dataset was constructed with these individuals that included the predicted probability of cluster assignment for any sampled patient for the Stacked model in the case of the *Unsupervised Approach* and the p-ORM in the case of the *Supervised Approach*. Linear regression was performed on each of these features using the electrophysiological score associated with each variant as the response feature. For the explanatory feature, an expected severity score for each patient was calculated by multiplying the probabilities of the patient being cluster U1/S1, cluster U2/S2, and cluster U3/S3 by one, two, and three, respectively.

Clinical validation was deemed necessary for the *Supervised Approach* as the cluster assignments for this approach follow clinical conventions, while the *Unsupervised Approach* follows an alternative decision-making process. A new dataset (*n*=91) was constructed using individuals reported in Johannesen et al., 2022, with features that correspond to the prediction features of the p-ORM. Individuals from this dataset were cluster S1 if their phenotype was reported as benign familial infantile epilepsy (BFIE) or infantile epilepsy (IE), cluster S2 if reported as DEE with mild-moderate ID, and cluster S3 if reported as DEE with Severe ID. To convert the categorical variable of ID into a continuous variable as DQ, severe ID was set to have a DQ of 40, moderate ID a DQ of 67.5, mild ID a DQ of 90, and normal a DQ of 100. Additionally, seizure freedom was inferred if the patient was classified as BFIE or IE. Using the p-ORM trained in the *Supervised Approach,* these 91 individuals were predicted as cluster S1, cluster S2, or cluster S3 and a confusion matrix was constructed to assess performance with error as the primary assessment feature.

### Data and code availability

All coding was completed in RStudio version 4.2.2 and de-identified datasets and markdowns for dataset construction, expansion, analysis, and model validation are archived on Zenodo (doi: 10.5281/zenodo.8336484) (Bischl et al., 2016; Bischl et al., 2018, 2020; Fox and Hong, 2010; Lunardon et al., 2014; OpenML, 2020; Probst et al., 2017; Venables and Ripley, 2002; Wright and Ziegler, 2017).

### Ethics

This study was approved by the University of Arizona Institutional Review Board (#1603487278) and all caregivers of individuals with SCN8A-related disorders consented to participate prior to providing their filling out the International SCN8A Patient Registry.

## Supplementary Material

10.1242/biolopen.060286_sup1 Supplementary information

Table S1. Justification for variant inclusion in this study. Each patient’s variant was verified as being pathogenic or likely pathogenic by cross-referencing patient genetic report, the ClinVar database, and SCN8A literature. When variant was unable to be verified using these methods, the ACMG guidelines [27] on classifying a variant as pathogenic or likely pathogenic were followed. Justification for variants being classified as GOF is included for each variant, using either known electrophysiological studies or the LOF Classifier [16]. In instances where a patient was unable to be verified using these methods, justification for being GOF is included based on clinical features and response to medications.
