# Supplementary material for: Machine learning models reveal distinct disease subgroups and improve diagnostic and prognostic accuracy for individuals with pathogenic SCN8A gain-of-function variants
Source: Biol Open. 2024 Apr 24;13(4):bio060286. doi: 10.1242/bio.060286 (PMC11070785; doi:10.1242/bio.060286)
Supplement: Supplementary information [file biolopen-13-060286-s1.pdf]

Biology Open (2024): doi:10.1242/bio.060286: Supplementary information

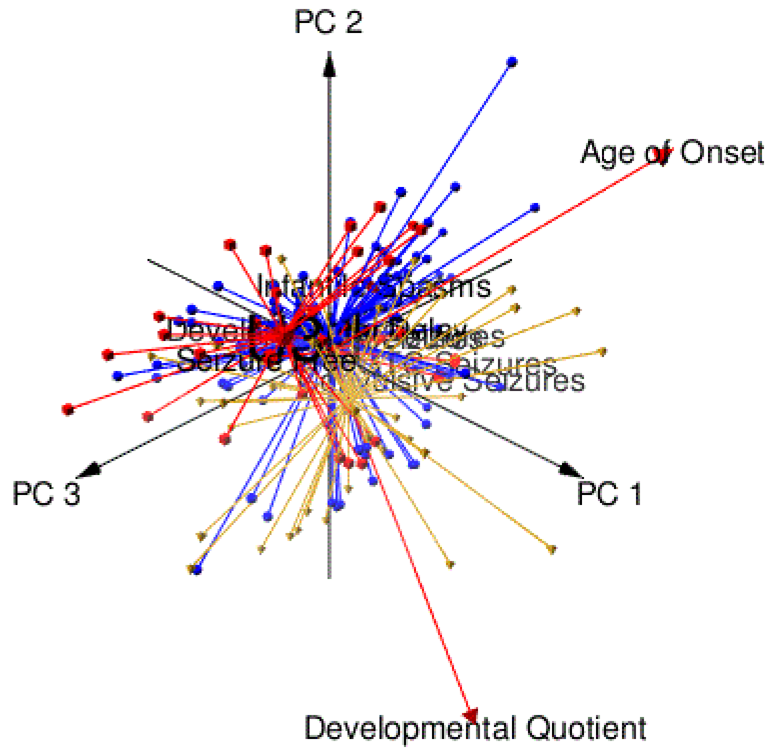

Fig. S1. 3-dimensional principal component plot of *Unsupervised Approach.* Cluster U3 (*green*), U2 (*blue*), and U1 (*yellow*) are shown with contribution from the top 5 features as vectors.

Biology Open • Supplementary information

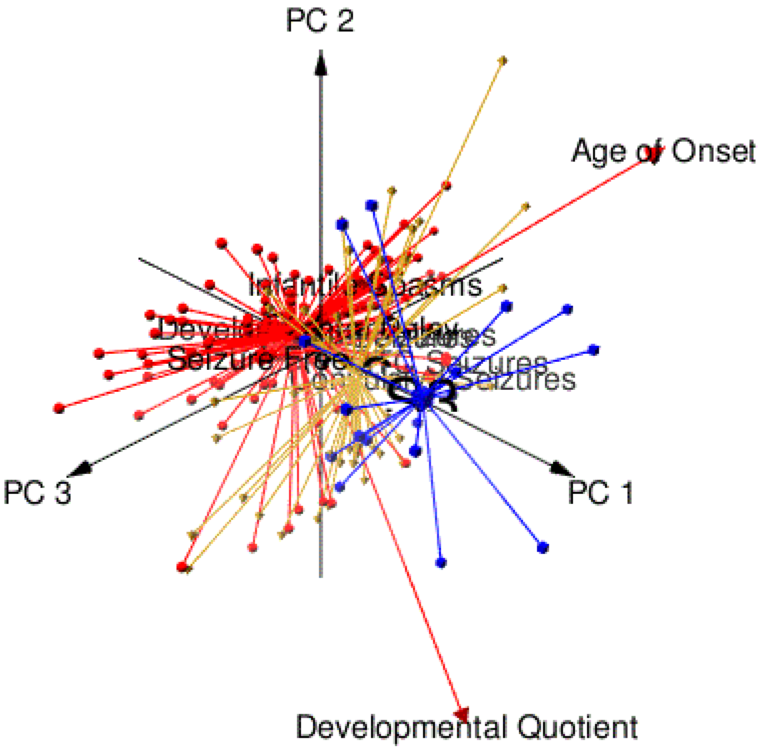

Fig. S2. 3-dimensional principal component plot of *Supervised Approach.* Cluster S3 (*green*), S2 (*blue*), and S1 (*yellow*) are shown with contribution from the top 5 features as vectors.

Table S1. Justification for variant inclusion in this study. Each patient's variant was verified as being pathogenic or likely pathogenic by cross-referencing patient genetic report, the ClinVar database, and *SCN8A* literature. When variant was unable to be verified using these methods, the ACMG guidelines [27] on classifying a variant as pathogenic or likely pathogenic were followed. Justification for variants being classified as GOF is included for each variant, using either known electrophysiological studies or the LOF Classifier [16]. In instances where a patient was unable to be verified using these methods, justification for being GOF is included based on clinical features and response to medications.

Available for download at
https://journals.biologists.com/bio/article-lookup/doi/10.1242/bio.060286#supplementary-data
